# Atmospheric Pressure

**Published:** 1851-01

**Authors:** James Fleming

**Affiliations:** Harrisburg, PA.


### ATMOSPHERIC PRESSURE.

BY JAMES FLEMING, M.D., HARRISBURG, PA.

There are some difficulties which lie in the way of our proceeding for the insertion of entire substitutes upon the above principle, which appear to be quite overlooked. Some of these I consider of very great importance; especially one, which I will first mention, and which lies at the foundation of the whole matter. It consists in the fact that a substance which is capable of taking a true impression of the gum, cannot be removed therefrom without more or less *distortion*, unless atmospheric pressures be taken off; or, in other words, the *vacuum* which has thus been created be destroyed. I have nowhere seen a plan proposed which was directly intended to obviate this difficulty. It is true that *plaster*, if it be good, and kept in the mouth a sufficient length of time, (a proceeding which is not very agreeable to the patient, to say the least of it,) will harden sufficiently to allow of its removal with but little if any *swagging*. But how can it be started, if it be perfectly *true*, without first moving it and altering its face sufficiently to take off at least a part of the pressure? For be it remembered that fifteen pounds to the square inch is no trifle to overcome; and if there is not a yielding somewhere, the effect upon the gum itself would be pretty severe. It is obvious that if the plate, which is to have the exact form of the impression, is to be sustained with any considerable degree of firmness, the impression itself will be even more firmly held, if there be no yielding any where. I am persuaded that a *perfect* impression can never be had, without taking off, in some way, atmospheric pressure, so as to allow of its easy withdrawal.

I have, for five or six years, been in the habit of adopting a very simple plan for obviating the above difficulty. And I believe that any one who will once test its utility, will not again omit using some means of the kind for the purpose alluded to. A few days ago I was trying a plate in a lady's mouth, which I had just prepared. After I had directed her to exhaust the air from under it, by suction, the effort which it took to remove it quite alarmed her, and she asked me, in all sincerity, "Is there any danger of its getting so fast in my mouth that I can't get it out?" This plate was prepared without an *air chamber*. Of this, however, I shall hereafter speak.

*To take the impression of the mouth*, I consider *beeswax*, when well managed, the very best material that can be used. I have seen it stated that it will sometimes stick to the mouth. This, I apprehend, is a mistake. It should not of course be used too soft. And it is much better to warm it at a fire, or over a spirit lamp, than in hot water. As to its *sliding* over the mouth, the difficulty is very easily obviated. If firm and steady pressure be made *directly against the palital arch*, the wax will receive the exact impression of that part of the mouth. Having selected a suitable wax-holder, (of which I have a variety, both in size and depth,) I place in it the wax, suitably prepared, and immediately perforate it through an opening previously made in the *holder* towards the center of the palatal arch, and place therein a *tube*, which is barely long enough to extend through, both ways. When the impression is being made, this tube will press downwards, and will leave a complete opening through the whole, for the admission of air, so that it can be removed without any difficulty and without distortion. Before doing so, however, it should be firmly held with one hand, while with the other, pressure is gently made all round the margin. It should then be moved in a direction straight off from the gum, starting it first in front. The sides of the holder should always be high enough to guard the impression from injury, in withdrawal from the mouth.

Having thus obtained a perfect impression of the mouth, there is another fact which must not be lost sight of at this stage of the proceeding, in order to obtain a *true model* therefrom. Wax will contract in cooling; and as it will be of different thickness at different points, after the impression is made, so will the degrees of contraction be varied over its surface. Hence, to prevent this effect, I make my *cast* at once, mixing the plaster with *tepid* water. In this way, I think there can be a nearer approach made to a perfect model of the mouth, than can be obtained in any other way.

As to the contraction which takes place in the *metal castings* I think it is, perhaps, an advantage. The plate will be somewhat expanded by the heat of the mouth; but what is still more worthy of consideration, there is generally more or less absorption of the gum for a while after it has been inserted. And in this process, there is an adaptation of its surface to the face of the plate.

In *swedging* up the plate, great care should be taken to go no further than is necessary to bring it to the exact form of the model; as any *stretching* beyond this point will be certain to impair the fit.

*Central and Lateral Air Chambers.*—Although I have, until very recently been quite in the habit of using these auxiliaries, as they are supposed to be, for the insertion of upper sets, yet I must say it was never without some misgivings as to the correctness of the principle. If a plate be perfectly fitted to the whole arch and ridge, it needs no such assistance to make it adhere firmly. Indeed, the only effect of them appears to be to destroy the *equality* of pressure, and thus *to take away much from the comfort of the wearer*, besides lessening, perhaps, the amount of adhesive power. The plan which I have most recently adopted, and which has given me more satisfaction than any other, is to place in the plaster model, before making my zinc cast, a piece of very thin wax paper, cut into such a shape as to extend over what would be the *most solid portion* of the palatal arch, taking care not to come, at any place, to the edge of the plate. It should be as thin as card paper, and pressed firmly down upon the cast; the object being to allow of the ultimate contact of the entire plate with the gum. It was in this way that I prepared the plate alluded to above.—*Dental News Letter.*

